# Recognizing the Operating Hand and the Hand-Changing Process for User Interface Adjustment on Smartphones †

**DOI:** 10.3390/s16081314

**Published:** 2016-08-20

**Authors:** Hansong Guo, He Huang, Liusheng Huang, Yu-E Sun

**Affiliations:** 1 School of Computer Science and Technology, University of Science and Technology of China, Hefei 230000, China; guohanso@mail.ustc.edu.cn (H.G.); lshuang@ustc.edu.cn (L.H.); 2 School of Computer Science and Technology, Soochow University, Soochow 215000, China; 3 School of Urban Rail Transportation, Soochow University, Soochow 215000, China; sunye12@suda.edu.cn; 4 School of Computer and Software, Nanjing University of Information Science and Technology, Nanjing 210000, China

**Keywords:** operating hand recognition, hand-changing process detection, user interface adjustment, smartphone, touchscreen, accelerometer and gyroscope, supervised classification

## Abstract

As the size of smartphone touchscreens has become larger and larger in recent years, operability with a single hand is getting worse, especially for female users. We envision that user experience can be significantly improved if smartphones are able to recognize the current operating hand, detect the hand-changing process and then adjust the user interfaces subsequently. In this paper, we proposed, implemented and evaluated two novel systems. The first one leverages the user-generated touchscreen traces to recognize the current operating hand, and the second one utilizes the accelerometer and gyroscope data of all kinds of activities in the user’s daily life to detect the hand-changing process. These two systems are based on two supervised classifiers constructed from a series of refined touchscreen trace, accelerometer and gyroscope features. As opposed to existing solutions that all require users to select the current operating hand or confirm the hand-changing process manually, our systems follow much more convenient and practical methods and allow users to change the operating hand frequently without any harm to the user experience. We conduct extensive experiments on Samsung Galaxy S4 smartphones, and the evaluation results demonstrate that our proposed systems can recognize the current operating hand and detect the hand-changing process with 94.1% and 93.9% precision and 94.1% and 93.7% True Positive Rates (TPR) respectively, when deciding with a single touchscreen trace or accelerometer-gyroscope data segment, and the False Positive Rates (FPR) are as low as 2.6% and 0.7% accordingly. These two systems can either work completely independently and achieve pretty high accuracies or work jointly to further improve the recognition accuracy.

## 1. Introduction

**Figure 1:**
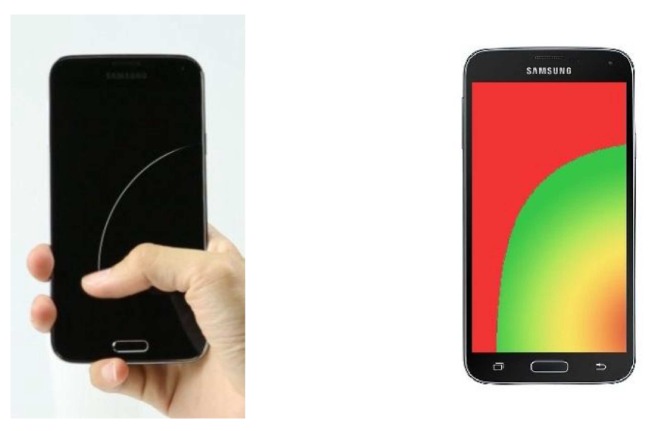
Limited touching range of the thumb on the Samsung Galaxy S5.

As technology advances, smartphones with abundant built-in sensors are becoming more and more ubiquitous in our daily lives, which stimulates the blooming of smartphone sensing research, such as healthcare, localization and human computer interaction and makes our lives more efficient, more intelligent and more enjoyable. In this paper, we also focus on this field. The smartphone operating habits are different for left-handed and right-handed people, especially the requirements of the user interfaces. In addition, some users change operating hands frequently. The result of our investigation about the dominant hand when operating smartphones by 500 randomly-selected students from the University of Science and Technology of China shows that 34% of them usually operate the smartphones with the left hand, 50% usually with the right hand and almost 16% operate the smartphones utilizing the right or left hand with the same frequency. This problem was not that severe previously since the sizes of smartphone screens were small. However, things are worse as the sizes have become larger in recent years. For example, the screen size of iPhone 6 has already reached 4.7 inches [1], while the screen size of iPhone 4 is only 3.5 inches [1], and the screen sizes of the Nokia Lumia 930 and Samsung Galaxy S5 have already reached 5.0 and 5.1 inches, respectively [2,3]. Users’ visual experiences are improved with the continuous increase of smartphone screens sizes; meanwhile, the single hand operability of smartphones is significantly getting worse, as depicted in Figure 1, especially for female users.

**Figure 2:**
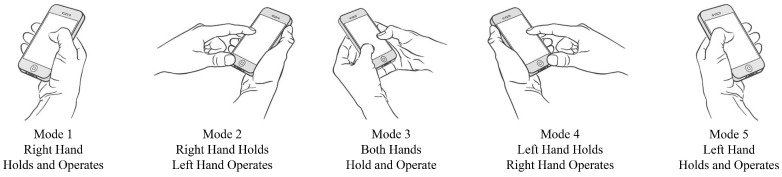
Five smartphone operation modes.

To address this challenge, we propose, to the best of our knowledge, the first scheme for detecting the current operating hand from touchscreen traces only, which is covered in [4] and the first scheme for detecting the hand-changing process. As for the scheme for detecting the current operating hand, we divide smartphone operation modes into five main categories based on numerous observations of users’ daily lives, as shown in Figure 2. User interfaces of large-screen smartphones can be adjusted for each particular mode, especially for Mode 1 and Mode 5, whose touching ranges are limited. The accuracy of detecting the current operating hand by our proposed scheme is 94.1% when deciding with a single trace only. As for the scheme for detecting the hand-changing process, after recognizing that the user is passing the smartphone from the right hand to the left hand, or vice versa, the user interfaces of large-screen smartphones can be adjusted accordingly, to make smartphones easy to operate by the left hand or the right hand. The accuracy of detecting the hand-changing process by our proposed scheme is 93.7% when deciding with a single segment only. These two schemes proposed in this paper can both work completely independently and achieve pretty high accuracies or they can also work jointly to further improve the recognition accuracy and reduce the false user interface adjustments ulteriorly, which depends on the user’s choice, after synthetically considering the accuracy, energy consumption and system overheads. This part is easy and can be flexibly arranged, so it is beyond the scope of this paper.

### 1.1. Motivations

Following are some common scenarios in smartphone (with large screen) users’ daily lives.

- People are operating smartphones with the right (or left) hand, but the buttons they want to press are on the top left (or right) corner. Since smartphone screens are large, they have to try their best to reach these buttons. They will be forced to use both hands if these buttons are too far away, because almost all of the user interfaces today are fixed, which is inconvenient when the other hand is busy doing other things, such as eating, carrying heavy loads, holding the handle of a metro and driving a car.
- People frequently use the input method or the dialing keyboard to interact with smartphones, and these two approaches usually occupy the entire width of the smartphone screen nowadays. Therefore, they have to use both hands to input if the smartphone screens are large. User experience will be greatly improved if the smartphone can dynamically detect the current operating hand and then shrink the area of the input method or the dialing keyboard proportionally, as well as automatically let them gather on the side of this hand; because users can complete the whole input process with a single hand, even if the smartphone screens are very large.
- There are numerous kinds of buttons on the smartphone user interface, and some of them are sensitive or even destructive; for example, the send button for SMS, the dislike button for social software, such as Facebook, and the delete button of the photo album (e.g., in the Android 5.1.1 Operating System). A series of serious consequences may be caused if the user accidentally touches these buttons and is completely unaware of that. Therefore, these buttons should be placed at specific positions (the red area in Figure 1) of the smartphone user interface that are4 closely related to the current operating hand. Then, the users need to make some effort to reach these buttons if they really want to, so a casual touching event will never happen.
- There are three buttons at the bottom of most Android-based smartphones today, which are the back button, the home button and the menu button, respectively. The use of the back button is more frequent than the menu button for almost all users in our investigation. However, as far as we know, the positions of these three buttons on the Android-based smartphone user interface are all fixed nowadays. One more reasonable approach is putting the back button at the position that is easiest to touch among the three positions according to the current operating hand.

## 2. Recognition of the Current Operating Hand from Touchscreen Traces

In this section, we introduce our first system in detail, namely the implementation and evaluation of our proposed system for recognizing the current operating hand, from touchscreen traces.

### 2.1. System Overview

**Figure 3:**
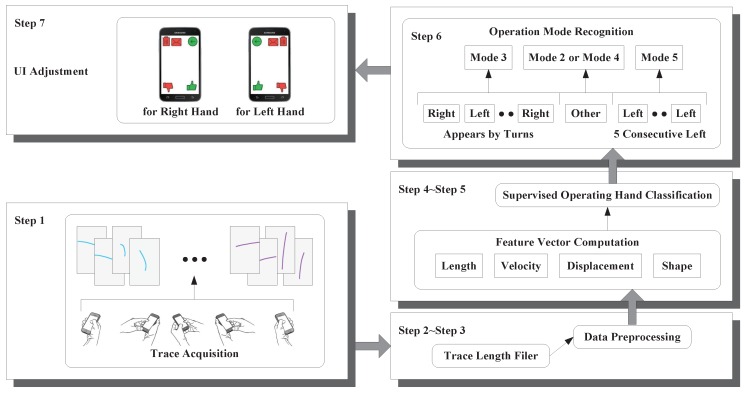
System overview of recognizing the current operating hand.

Here, we provide a system overview of our current operating hand recognition scheme as shown in Figure 3. The first step is the acquisition of touchscreen trace data from the smartphone touchscreen, which are organized in the form of a series of coordinate data with the corresponding timestamp at every sampling point. There is a length filter in the second step. Traces are delivered to this filter where the length of every trace will be compared to a certain threshold. Then, our scheme discards traces whose lengths are below this threshold, because these traces are too short to carry enough useful information, and features extracted from these traces provide a poor degree of distinction. Our scheme preprocesses the coordinate data of touchscreen traces in the third step in order to improve the accuracy of feature vector computation. In the fourth step, our scheme computes a feature vector consisting of features that can be utilized to recognize the current operating hand. Then, our scheme constructs a supervised classifier called Random Forest (RF), which outputs the recognition result Left,Right,Other for the current operating hand (finger), using features extracted in the fourth step. In the sixth step, our scheme recognizes the current operation mode (as shown in Figure 2) according to the continuous sequence of supervised operation hand classification results within a previous period of time. Our scheme recognizes user’s current operation mode as Mode 5 or Mode 1 when the results’ sequence contains at least *n* consecutive Left or *n* consecutive Right, respectively. *n* is a predefined variate that represents the tradeoff between the response time of our scheme and the recognition accuracy, and its value is five in this paper. Otherwise, if Left and Right appear in turn in the results’ sequence, then user’s current operation mode will be judged as Mode 3 by our scheme. Our scheme believes that the user is operating the smartphone with Mode 2 or Mode 4 when the operation hand classification result is Other. In the last step, there are many things the smartphone can do according to the operation mode recognition result, and the most relevant one is adjusting the user interface for the user if the recognition result is Mode 1 or Mode 5. 

### 2.2. Utilizing Pattern Recognition Algorithms for Recognition

In this part, we present the details when utilizing pattern recognition algorithms for the recognition in our first system.

#### 2.2.1. Computation of the Feature Vector

We introduce features that are selected to distinguish the current operating hand, as shown in Table 1.

**Figure 4:**
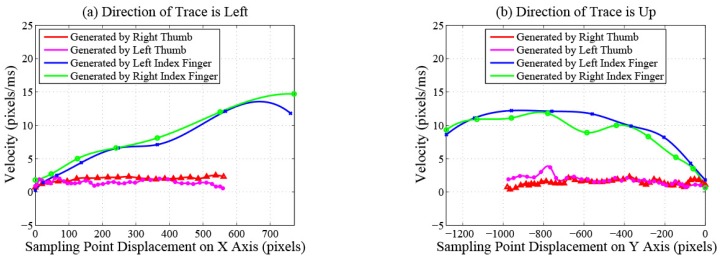
Velocity magnitude of each sampling point.

**Figure 5:**
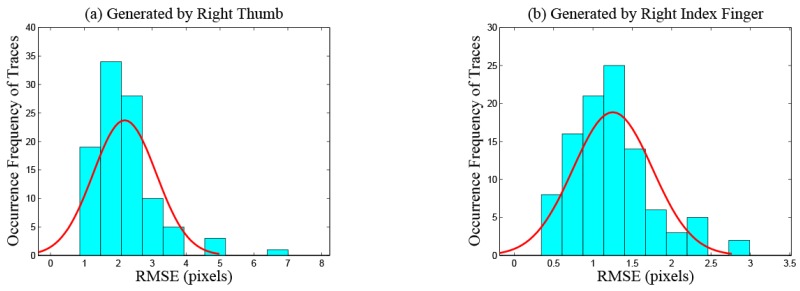
Root mean squared errors of 200 touchscreen traces.

**Figure 6:**
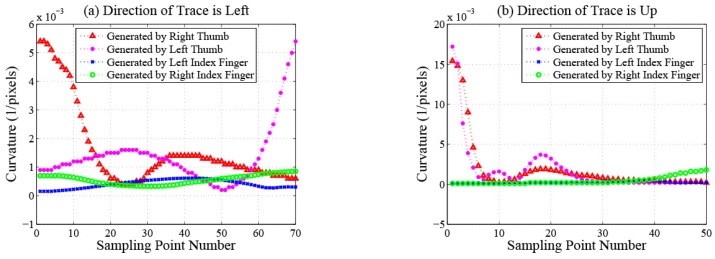
Curvature magnitude of each sampling point.

- Length features. Total length.
- Velocity features. Maximum and average velocity. Standard deviation of velocity. The changing process of the velocity on a trace contains two phases: acceleration and deceleration. The durations of acceleration processes are different between traces generated by index fingers and thumbs because of the different lengths of the traces. The deceleration processes of traces generated by index fingers are shorter because these traces often slide out of the touchscreens directly without deceleration. Therefore, the velocity features of traces generated by index fingers and thumbs are different. Figure 4 plots the change of the velocity magnitude with displacement on traces performed by different fingers of the same participant. The directions of traces are left in Figure 4a and up in Figure 4b. We can observe that, for this participant, velocity features can be utilized to distinguish traces generated by different fingers.
- Displacement features.
  Total and maximum X-displacement.
  Total and maximum Y-displacement.
- Shape features. In order to describe the shape features of traces quantitatively and accurately, we carry out curve fitting on discrete sampling points of every trace using a quartic polynomial, which is a tradeoff among fitting precision, computational complexity and the degree of distinction on traces generated by different fingers.
  Root mean squared error. This feature measures the smooth degree of traces. Index fingers are more flexible and have larger touching ranges than thumbs. When operating smartphones, index fingers exert less pressure on touchscreens and receive less friction resistance, so traces generated by index fingers are always smoother than those generated by thumbs. The root mean squared error of trace $T_{i}$ is calculated as:
  $$\mathrm{Root}\;\mathrm{Mean}\;\mathrm{Squared}\;{\mathrm{Error}}_{i}=\sqrt{\frac{1}{n_{i}}\sum_{j=1}^{n_{i}}{\left(y_{ij}-{\hat{y}}_{ij}\right)}^{2}}$$
  where $y_{ij}$ indicates the true *y* coordinate value of the *j*-th discrete sampling point on the trace $T_{i}$ and ${\hat{y}}_{ij}$ indicates the predicted *y* coordinate value of the *j*-th discrete sampling point calculated by carrying out curve fitting on the trace $T_{i}$. $n_{i}$ indicates the number of sampling points on the trace $T_{i}$. Figure 5 plots the distribution of the root mean squared errors from 200 traces generated by the right thumb (as shown in Figure 5a) and the right index finger (as shown in Figure 5b), respectively, of the same participant. The number of each kind of traces is 100, and all directions of the traces are right. We can observe that, for this participant, the values of the root mean squared error feature are different among traces generated by different fingers.
  Maximum and average curvature. These two features measure the curvature degree of traces. Users slide on touchscreens exploiting the most effortless approach unconsciously. When users operate smartphones with thumbs, restricted to the limited touching ranges, most of the traces generated are curves whose centers are on the same side as the operating hand. The touching ranges of index fingers are significantly larger, which produces straighter traces. The curvature at the *j*-th sampling point of trace $T_{i}$ is calculated as:
  $${\mathrm{Curvature}}_{\mathrm{ij}}=\frac{f_{i}^{\prime \prime }\left(x_{ij}\right)}{{\left(1+f_{i}^{\prime }{\left(x_{ij}\right)}^{2}\right)}^{3/2}}$$
  where $f_{i}\left(x\right)$ indicates the fitting curve function of trace $T_{i}$ and $f_{i}^{\prime }\left(x\right)$, $f_{i}^{\prime \prime }\left(x\right)$ indicate the first order derivative and the second order derivative of $f_{i}\left(x\right)$, respectively. $x_{ij}$ indicates the *x* coordinate value of the *j*-th discrete sampling point on the trace $T_{i}$. Figure 6 plots the curvature magnitude at every sampling point on traces performed by different fingers of the same participant. The directions of traces are left in Figure 6a and up in Figure 6b. We can observe that, for this participant, curvature features can be utilized to distinguish traces generated by different fingers.
  Curve convex orientation. This feature measures the curve’s convex orientation, which can be very useful in distinguishing traces generated by the left thumb and the right thumb. To calculate the curve convex orientation of trace $T_{i}$, first, we randomly choose a sampling point that is close to the middle of the trace. Second, we construct two vectors, which are from this sampling point to the first and the last sampling point, respectively. Then, we calculate the cross product between these two vectors.
  $$\vec{z_{i}}=det\left|\begin{array}{ccc} \;\vec{x} & \vec{y} & \vec{z}\;\;\\ \;x_{i1}-x_{ik} & y_{i1}-y_{ik} & 0\;\;\\ \;x_{in_{i}}-x_{ik} & y_{in_{i}}-y_{ik} & 0\;\; \end{array}\right|$$
  where $\left(x_{i1},y_{i1}\right)$ and $\left(x_{in_{i}},y_{in_{i}}\right)$ indicate the coordinates of the first and the last sampling point of trace $T_{i}$, respectively. $\left(x_{ik},y_{ik}\right)$ indicates the coordinates of the randomly chosen sampling point. Finally, the curve convex orientation of trace $T_{i}$ is calculated as:
  $$\mathrm{Curve}\;\mathrm{Convex}\;{\mathrm{Orientation}}_{i}=sgn\left\{\left(y_{in_{i}}-y_{i1}\right)\vec{z_{i}}\cdot \vec{z}\;\right\}$$
  where sgn is the sign function.

### 2.3. Evaluation

In this part, we report the performance for recognizing the current operating hand of our first system, when utilizing pattern recognition algorithms.

#### 2.3.1. Recognition Performance of Pattern Recognition Algorithms

Here, we present the results of our experiments, which consist of six parts. In the first part, we demonstrate the computation time our scheme spends on feature vector extraction. Then, we compare the differences in classification performance among five common classifiers in the second part. In the following two parts, we study the impact of the number of training samples and the number of trees in Random Forest (RF) on classification performance, respectively. Finally, we report the evaluation results of our scheme.

According to the direction (displacement characteristics), we divide all touchscreen traces into four categories: right, left, up and down. There are 14 participants in our experiment. We ask every participant to generate traces in each direction with the right thumb, left thumb, right index finger and left index finger successively in the procedure of data collection. However, in the actual use of our scheme, the user could only generate traces in a subset of four directions if traces with directions in the complementary set do not appear frequently in his or her daily smartphone operation. That is to say, the user can make a tradeoff between the convenience of the training traces acquisition process and response time, because our scheme will not respond to traces for which the directions are not in the subset above. In this paper, we request all participants to generate traces in all directions in order to adequately test the classification performance of our scheme on multifarious traces. Therefore, there will be 16 trace subsets in total.

##### Computation Time of the Feature Vector

Table 2 shows the computation time (Avg. ± SD) of every feature vector from 100 touchscreen traces. We can see that the RMSE, the maximum curvature and the average curvature are the most time-consuming features, and all features can be calculated within about 17 ms.

##### Multi-Class Classification Algorithms

In this paper, we use precision, recall, F1, FPR and AUC to measure classification performance, and AUC is the area under the Receiver Operating Characteristic (ROC) curve.

**Figure 7:**
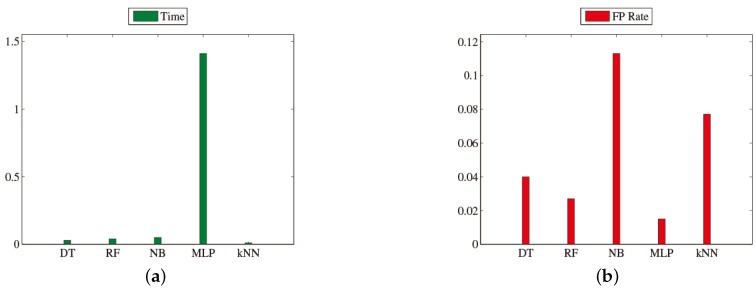
(**a**) Total time and (**b**) FPR of different classifiers.

In this part, we construct five different classifiers [5,6], which are Decision Tree (DT) [7,8], Random Forest (RF) [9,10], Naive Bayes (NB) [11,12], Multi-Layer Perceptron (MLP) [13,14] and k-Nearest Neighbors (k-NN) [15,16], and compare their classification performance on our touchscreen traces data. The number of traces in each training subset and test subset is 40 and 10, respectively, in this part, so the total number of traces in the training set and the test set is 640 and 160. Table 3 shows the classification performance, and Figure 7 shows the total time (training time + test time) and FPR of these five classifiers. The *x*-axes of Figure 7a,b are different classifiers. The *y*-axes of Figure 7a,b are total time and FPR, respectively.

We can make two observations here. Firstly, on the one hand, MLP gives the best classification performance, which achieves 96.9% precision and 1.5% FPR, and RF gives the second best classification performance, which achieves 95.6% precision and 2.7% FPR. On the other hand, NB achieves the worst classification performance, and k-NN is also not good enough. Second, as shown in Figure 7, the total time of MLP is 1.41 s, but the total time of RF is only 0.04 s (almost 0 ms for every trace). The former is 35-times as long as the latter. After considering all factors above, we finally choose RF as our classifier in this paper, which is extremely effective for datasets containing many redundant features. As mentioned before, the time consumption of feature extraction is about 17 ms. The time consumption of the remaining steps is too short to measure. Therefore, for every trace, the whole recognition process can be accomplished within about 17 ms.

##### Impact of the Number of Training Samples

**Figure 8:**
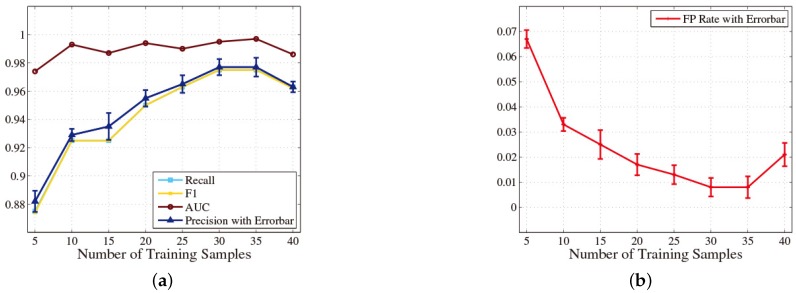
(**a**,**b**) Impact of the number of training samples.

In this part, we indicate the impact of the number of training samples on the classification performance. The number of training samples is a significant parameter in the construction process of a classification model. On the one hand, if the number of training samples is too large, the workloads of training traces’ acquisition will become onerous for users. Moreover, although the training error of the classification model is low, the generalization error may be high sometimes, because the classification model is over fitting, which will lead to poor classification performance eventually. On the other hand, if the number of training samples is too small, the classification model will be under fitting, which also leads to inaccurate classification results. The number of traces in each test subset is five, so the total number of traces in the test set is 80 in this part. Figure 8 shows the change of classification performance with the increase in the number of traces in each training subset.

We can make two observations here. Firstly, the classification performance is significantly improved with the increase in the size of each training subset from five to 10. As shown in Figure 8, the precision increases from 88.2% to 92.9%, and the FPR drops from 6.7% to 3.3%. This is because the number of traces in the training set is larger than the test set for the first time. Secondly, the RF achieves the optimal classification performance when there are 30 traces in each training subset. Figure 8 shows that the precision can be as high as 97.7%, and the FPR achieves 0.8% accordingly.

##### Impact of the Number of Trees

**Figure 9:**
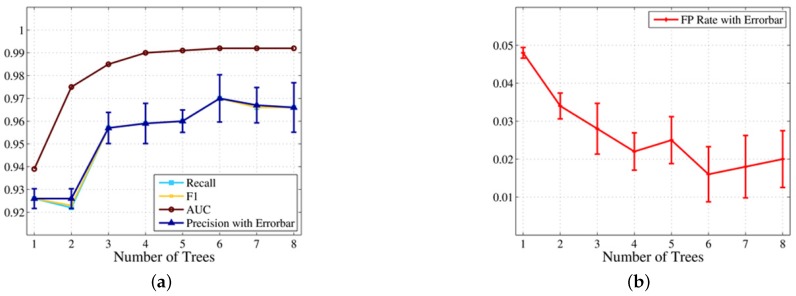
(**a**,**b**) Impact of the number of trees in RF.

In this part, we study the impact of the number of trees on the classification performance. The number of trees is another significant parameter in RF. On the one hand, if the number of trees is too large in RF, there will be many trees using features with a high degree of similarity, which makes the construction process of the classification model spend too much time on less useful work. On the other hand, if the number of trees is too small in RF, the combination of different features will not be sufficient, so the RF cannot output the optimal classification results. We conduct a five-fold cross-validation experiment on 16 touchscreen trace subsets, and each subset contains 50 samples. Figure 9 shows the change of the classification performance with the increase in the number of trees in RF.

We can make two observations here. Firstly, the classification performance is significantly improved with the increase of the number of trees in RF from two to thee. As shown in Figure 9, the precision increases from 92.6% to 95.7%, and the FPR drops from 3.4% to 2.8%. This is because an RF containing three trees reflects the advantages of ensemble classifiers compared to a single DT for the first time. Secondly, the RF achieves optimal classification performance when there are six trees. Figure 9 shows that the precision can be as high as 97.0%, and the FPR achieves 1.6% accordingly.

##### Evaluation

**Figure 10:**
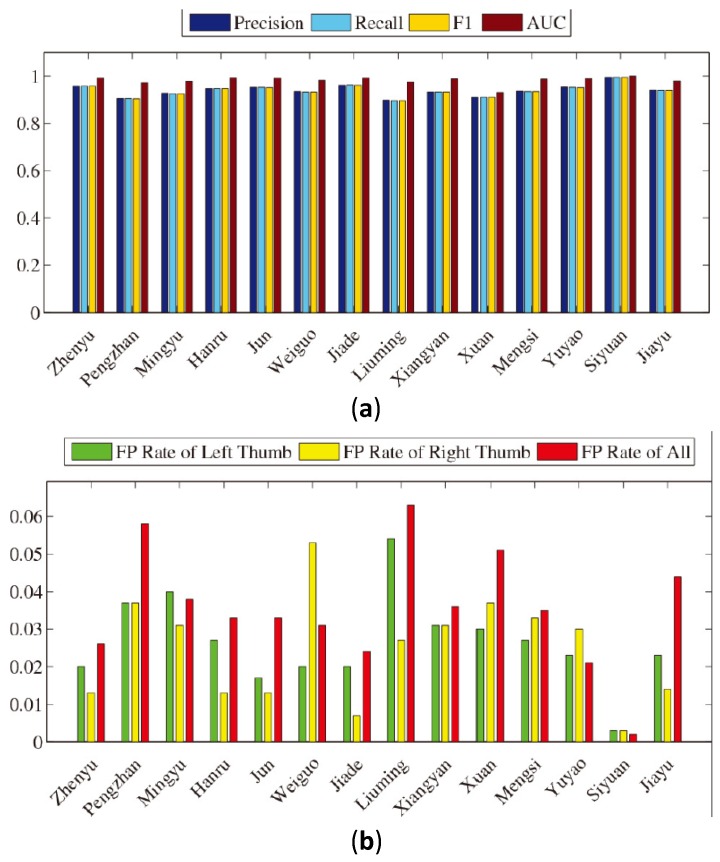
(**a**,**b**) Evaluation results of 14 participants.

In this part, we evaluate our scheme on two sets of 14 participants (seven male and seven female participants). We ask every participant to generate traces in four directions with the right thumb, left thumb, right index finger and left index finger, respectively, among which left (or right) traces are generated by some operations, such as navigating among main screen pages or viewing images, and up (or down) traces are generated by reading documents or browsing web pages. The number of traces in each subset is 25. Then, we conduct a five-fold cross-validation experiment on these traces. The average precision, TPR, FPR of the left thumb and FPR of the right thumb over all 14 participants turned out to be 94.1%, 94.1%, 2.7% and 2.4%, respectively, when deciding with a single trace only. Figure 10a,b shows the bar plots of the evaluation results of 14 participants.

## 3. Recognition of the Hand-Changing Process from the Accelerometer and Gyroscope Data

In this section, we describe in detail our second system, that is the implementation and evaluation of our proposed system for recognizing the hand-changing process, from accelerometer and gyroscope data.

### 3.1. System Overview

**Figure 11:**
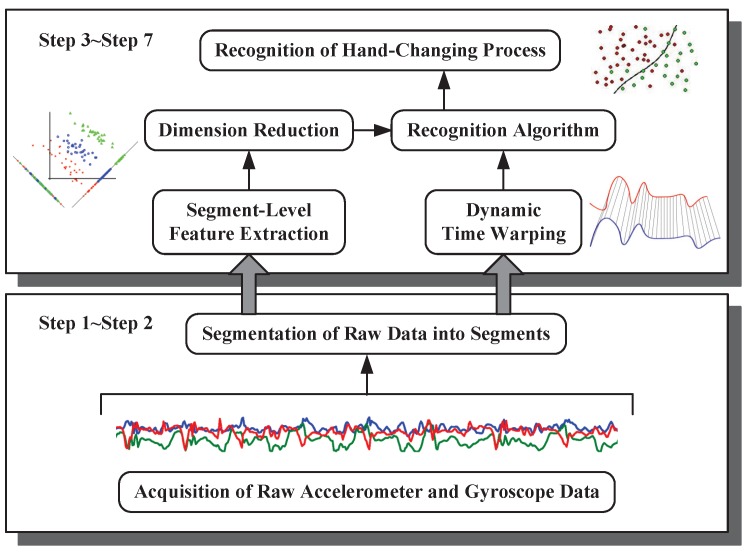
System overview of recognizing the hand-changing process.

In our hand-changing recognition process, as shown in Figure 11, some dedicated processing steps are performed [17,18,19]. The first step is the acquisition of raw accelerometer and gyroscope data from smartphones, which are organized in the form of triples (*x*-axis, *y*-axis and *z*-axis) with corresponding timestamps. Then, an end-points detection-based segmentation algorithm is exploited to extract the segments we are interested in from these accelerometer and gyroscope data time series. In the third step, we compute a feature vector consisting of the time domain, the frequency domain and statistics features, which contain important cues for distinguishing segments generated by various actions. In order to evaluate the sparseness, to maximize the synergies between different features, we conduct the dimension reduction in the fourth step. In this paper, we also exploit the performance of utilizing Dynamic Time Warping (DTW) distances as features to recognize the hand-changing process, as presented in the fifth step. Finally, we construct a recognizer, which outputs the recognition results, namely hand-changing processes or other actions using features extracted in the former step.

### 3.2. Activities of Daily Life

**Figure 12:**
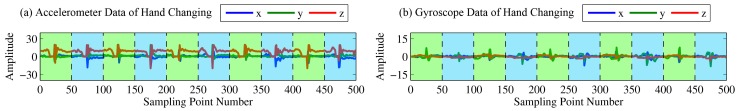
Accelerometer (**a**) and gyroscope (**b**) data of hand changing.

**Figure 13:**
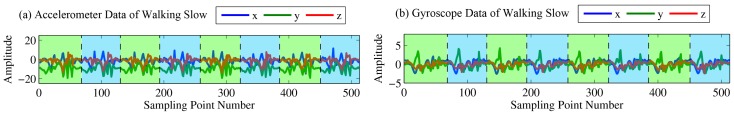
Accelerometer (**a**) and gyroscope (**b**) data of walking slowly.

**Figure 14:**
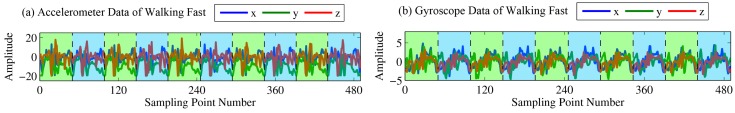
Accelerometer (**a**) and gyroscope (**b**) data of walking quickly.

**Figure 15:**
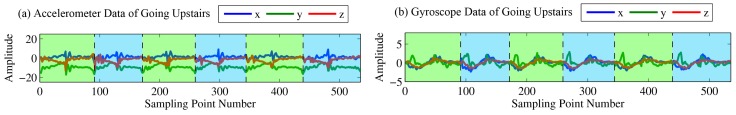
Accelerometer (**a**) and gyroscope (**b**) data of going upstairs.

**Figure 16:**
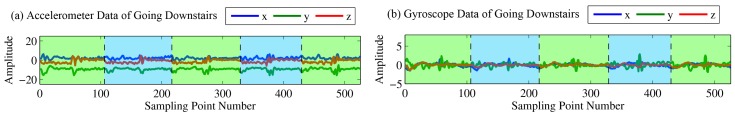
Accelerometer (**a**) and gyroscope (**b**) data of going downstairs.

**Figure 17:**
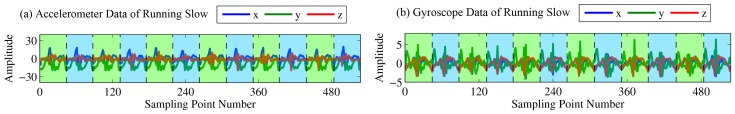
Accelerometer (**a**) and gyroscope (**b**) data of running slowly.

**Figure 18:**
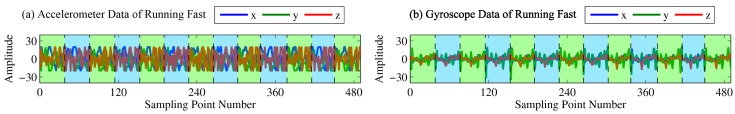
Accelerometer (**a**) and gyroscope (**b**) data of running quickly.

In this part, we illustrate several segments generated by hand-changing processes and activities of daily life. Whereafter, in Figure 12a,b, we plot the waveforms of the accelerometer and gyroscope data generated by hand-changing processes, including passing the smartphone from both the right hand to the left hand and the left hand to the right hand. In Figure 13a,b and Figure 14a,b, we plot the waveforms of the accelerometer and gyroscope data generated by walking slowly and quickly, respectively. In Figure 15a,b and Figure 16a,b, we plot the waveforms of the accelerometer and gyroscope data generated by going upstairs and downstairs, respectively. Finally, in Figure 17a,b and Figure 18a,b, we plot the waveforms of the accelerometer and gyroscope data generated by running slowly and quickly, respectively.

### 3.3. Segmentation Algorithm

#### End-Points Detection

Accelerometer and gyroscope data time series are divided into segments by our proposed segmentation algorithm. Sliding windows [20,21] and end-points detection [22,23] are two of the most popular segmentation methodologies. The sliding windows-based segmentation algorithm produces massive segments, the processing, storage and recognition of which will consume enormous energy, computing capabilities and memory, so the segmentation algorithm we utilized in this paper is based on end-points detection, which focuses on particular segments that we are interested in and filters out extraneous segments.

### 3.4. Utilizing Pattern Recognition Algorithms for Recognition

In this part, we provide the details when utilizing pattern recognition algorithms for recognition in our second system.

#### 3.4.1. Computation of the Feature Vector

Features can be considered as the abstractions of raw accelerometer and gyroscope data. Proper features can precisely represent the main characteristics of data segments. In this section, we calculate and manually refine a set of features from massive raw accelerometer and gyroscope data and then organize them in the form of a feature vector, which can be utilized as inputs to different pattern recognition algorithms to detect the hand-changing process from the activities of daily life. All features are divided into three categories, namely the time domain features, statistics features and frequency domain features, as shown in Table 4 and Table 5, respectively, among which the prefix X-, Y- or Z- indicates that this feature is extracted on the *x*-axis, *y*-axis or *z*-axis of the accelerometer or gyroscope data, the prefix AbsX-, AbsY- or AbsZ- indicates that this feature is extracted on the *x*-axis, *y*-axis or *z*-axis of the absolute values of the accelerometer or gyroscope data, and the prefix All- indicates that this feature is extracted on the combination of the *x*-axis, *y*-axis and *z*-axis of the accelerometer or gyroscope data; in other words, we calculate the value of $x^{2}+y^{2}+z^{2}$ at every sampling point of the accelerometer or gyroscope data.

- Time domain features. Time domain features focus on intuitive waveform characteristics, which can be obtained from data segments directly, so very small computational complexity and storage memory are required.
  Max, Mean, Min and range. These features describe the basic shape of accelerometer and gyroscope data segments and have been extensively exploited in various works, especially threshold-based algorithms.
- Statistics features. Statistics features capture the distribution characteristics of consecutive accelerometer and gyroscope sampling points.
  Kurtosis. This feature weighs how the amplitude decays near the extreme points, namely the peakedness and flatness. Larger kurtosis values indicate a more peaked distribution. The kurtosis of the accelerometer or gyroscope segment $S_{i}$ is calculated as:
  $${\mathrm{Kurtosis}}_{i}=\frac{n_{i}^{t}\sum_{j=1}^{n_{i}^{t}}{\left(s_{ij}-\bar{s_{i}}\right)}^{4}}{{\left(\sum_{j=1}^{n_{i}^{t}}{\left(s_{ij}-\bar{s_{i}}\right)}^{2}\right)}^{2}}$$
  where $s_{ij}$ indicates the *x*-axis, *y*-axis or *z*-axis value of the *j*-th sampling point in accelerometer or gyroscope segment $S_{i}$ and $\bar{s_{i}}$ indicates the mean *x*-axis, *y*-axis or *z*-axis value of all sampling points in accelerometer or gyroscope segment $S_{i}$. $n_{i}^{t}$ is the total number of sampling points in accelerometer or gyroscope segment $S_{i}$.
- Frequency domain features. Frequency domain features pay attention to the periodic nature. We transform the time series of accelerometer or gyroscope data into spectrum employing FFT (Fast Fourier Transform) in this paper. Centroid. This feature characterizes the barycenter of the frequency spectrum. The centroid of the accelerometer or gyroscope segment $S_{i}$ is calculated as:
  $${\mathrm{Centroid}}_{i}=\frac{\sum_{j=2}^{n_{i}^{f}}a_{ij}\log_{2}\frac{f_{ij}}{1000}}{\sum_{j=2}^{n_{i}^{f}}a_{ij}}$$
  where $n_{i}^{f}$ indicates the total number of frequency components and $a_{ij}$ indicates the amplitude of the *j*-th frequency component $f_{ij}$, in the frequency spectrum of the accelerometer or gyroscope segment $S_{i}$.
  DC amplitude. This feature denotes the amplitude of the DC component.
  Decrease. This feature weighs the decreasing degree of the frequency spectrum curve. The Decrease of the accelerometer or gyroscope segment $S_{i}$ is calculated as:
  $${\mathrm{Decrease}}_{i}=\frac{1}{\sum_{j=2}^{n_{i}^{f}}a_{ij}}\sum_{j=2}^{n_{i}^{f}}\frac{a_{ij}-a_{i1}}{j-1}$$
  Flux. This feature describes the stability of the frequency spectrum curve, in other words, how often the frequency spectrum curve of the accelerometer or gyroscope signal changes. The flux of the accelerometer or gyroscope segment $S_{i}$ is formally defined as:
  $${\mathrm{Flux}}_{i}=\sum_{j=2}^{n_{i}^{f}}{\left(a_{ij}-a_{i(j-1)}\right)}^{2}$$
  We choose the L2-norm, namely the Euclidean distance and the unnormalized spectrum when computing flux in this paper.
  Peak amplitude. This feature measures the max amplitude in the frequency spectrum. The peak amplitude of the accelerometer or gyroscope segment $S_{i}$ is calculated as:
  $$\mathrm{Peak}\;{\mathrm{Amplitude}}_{i}=a_{ij^{\prime }}$$
  $$j^{\prime }=\underset{j\in \left[2,n_{i}^{f}\right]}{arg max}|F_{j}\left(s_{i}\right)|$$
  where $|F_{j}\left(s_{i}\right)|$ indicates the amplitude of the *j*-th frequency component of the accelerometer or gyroscope segment $S_{i}$ after fast Fourier transformation and $s_{i}$ indicates the *x*-axis, *y*-axis or *z*-axis values of all sampling points in accelerometer or gyroscope segment $S_{i}$.
  Roll-off. This feature captures the frequency below which 75% of the total amplitudes of all frequency components is contained. The roll-off of accelerometer or gyroscope segment $S_{i}$ is calculated as:
  $$\mathrm{Roll}-{\mathrm{Off}}_{i}=\min\;j^{\prime },\;\text{subject to}:$$
  $$\sum_{j=1}^{j^{\prime }}a_{ij}\geq 0.75\sum_{j=1}^{n_{i}^{f}}a_{ij}$$
  Spread. This feature denotes the shape of the frequency spectrum, that is to say whether it is concentrated in the vicinity of its centroid or spread out over the frequency spectrum. The spread of the accelerometer or gyroscope segment $S_{i}$ is calculated as:
  $${\mathrm{Spread}}_{i}=\sqrt{\frac{\sum_{j=2}^{n_{i}^{f}}{\left(\log_{2}\frac{f_{ij}}{1000}-Centroid_{i}\right)}^{2}a_{ij}}{\sum_{j=2}^{n_{i}^{f}}a_{ij}}}$$

#### 3.4.2. Dimension Reduction of the Feature Vector

The feature vector is 54 dimensions. In order to evaluate the sparseness, to maximize the synergies between different features and then reduce the dimension of the feature vector, we explore Linear Discriminant Analysis (LDA) [24,25,26] and Principal Component Analysis (PCA) [27,28,29] in this paper.

### 3.5. Utilizing Dynamic Time Warping for Recognition

In this part, we give the details when utilizing DTW for recognition in our second system.

#### Preliminaries about DTW

**Figure 19:**
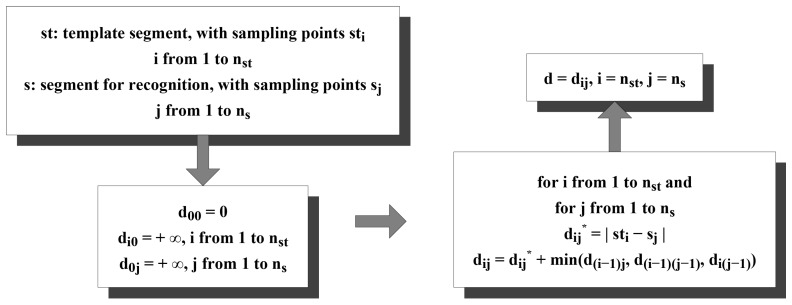
Computational process of the DTW.

DTW is a famous algorithm to explore and find out the optimal alignment, then to measure the similarity between two given time series under certain restrictions, which has been applied to automatic speech recognition and several other application scenarios [30,31,32]. In this paper, we also innovatively exploit whether it is possible to calculate the DTW distances between a particular segment and every segment in the template library and utilize them as features for recognition. The detailed procedures are provided in Figure 19, Algorithms 1 and 2.
**Algorithm 1:** Calculate DTW distance set. 1: *T*: template library containing $n_{t}$ template segments. 2: **Input:** 3: *s*: segment for recognition, whose class is unknown. 4: **Output:** 5: *D*: DTW distance set containing $n_{t}$ DTW distances between *s* and each template segment. 6: **for** each template segment $st_{i}$ in *T*
**do** 7:  $d_{i}$ = dynamic time warping ($st_{i}$, *s*); 8:  add $d_{i}$ into *D*; 9: **end for**
**Algorithm 2:** Dynamic time warping. 1: **Input:** 2: st: template segment, with $n_{st}$ sampling points $st_{1}$ to $st_{n_{st}}$. 3: *s*: segment for recognition, whose class is unknown, with $n_{s}$ sampling points $s_{1}$ to $s_{n_{s}}$. 4: **Output:** 5: *d*: DTW distance between *s* and st. 6: normalize the amplitude of each sampling point in *s* into [-1, 1]; 7: normalize the amplitude of each sampling point in st into [-1, 1]; 8: $d_{00}$ = 0; 9: **for**
*i* = 1 to $n_{st}$
**do** 10:  $d_{i0}$ = +*∞*; 11: **end for** 12: **for**
*j* = 1 to $n_{s}$
**do** 13:  $d_{0j}$ = +*∞*; 14: **end for** 15: **for**
*i* = 1 to $n_{st}$
**do** 16:  **for**
*j* = 1 to $n_{s}$
**do** 17:   ${\tilde{d}}_{ij}$ = $|st_{i}-s_{j}|$; 18:   $d_{ij}$ = ${\tilde{d}}_{ij}$+ min$(d_{(i-1)j}$, $d_{\left(i-1)(j-1\right)}$, $d_{i(j-1)})$; 19:  **end for** 20: **end for** 21: *d* = $d_{n_{st}n_{s}}$;

### 3.6. Evaluation

In this part, we study the performance for recognizing the hand-changing process of our second system, when utilizing pattern recognition algorithms and DTW, respectively.

#### 3.6.1. Recognition Performance of Pattern Recognition Algorithms

Here, we provide the results of our experiments, including five parts. In the first part, we present our experimental dataset. Then, we compare the differences in recognition performance among five common recognizers in the second part. In the third part, we explore the performance of two dimension reduction algorithms. Finally, we demonstrate the time consumption of every step in the recognition process for every segment.

##### Experimental Dataset

As shown in Table 6, our experimental dataset contains 500 hand-changing processes performed by passing the smartphone from the left hand to the right hand, 500 hand-changing processes performed by passing the smartphone from the right hand to the left hand and 130,050 segments generated by all kinds of activities in seven days of daily life.

##### Multi-Class Recognition Algorithms

**Figure 20:**
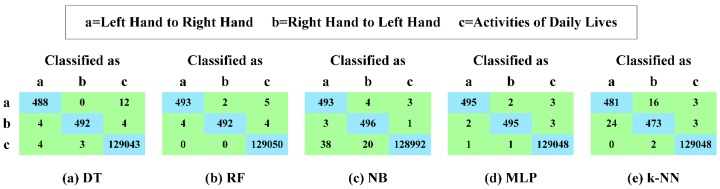
Confusion matrices of different recognition algorithms. (**a**) DT; (**b**) RF; (**c**) NB; (**d**) MLP; (**e**) k-NN.

**Figure 21:**
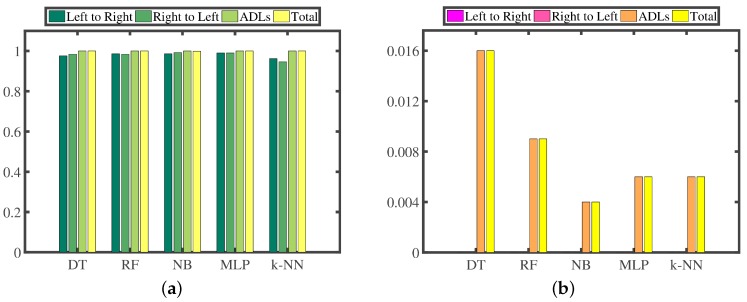
True positive rate (TPR) (**a**) and false positive rate (FPR) (**b**) of different recognition algorithms.

**Figure 22:**
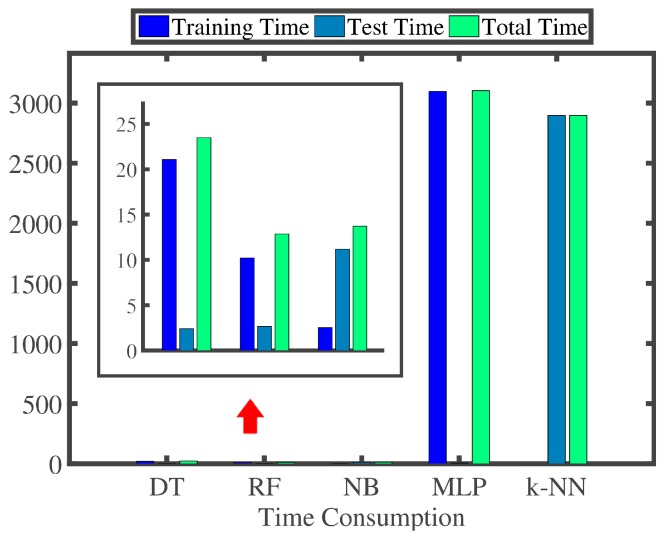
Total time consumed by different recognition algorithms.

We construct five multi-class recognizers based on different recognition algorithms [33,34,35,36,37,38,39,40], which are Decision Tree (DT), Random Forest (RF), Naive Bayes (NB), Multi-Layer Perceptron (MLP) and k-Nearest Neighbors (k-NN), respectively. Then, we conduct a series of 10-fold cross-validation experiments. Firstly, we randomly partition our experimental dataset into 10 mutually exclusive folds, and all folds each have an approximately equal size. Secondly, training and testing processes are performed 10 times, that is to say, in the *i*-th iteration, fold *i* is retained for testing, and the remaining nine folds are used for training. Figure 20 presents the confusion matrices; Figure 21a illustrates the TPR; Figure 21b highlights the FPR; and Figure 22 reports the time consumption for recognition on our experimental dataset of these five different recognition algorithms. The TPR and FPR are calculated as:

$$\mathrm{True}\;\mathrm{Positive}\;\mathrm{Rate}=\frac{n_{tp}}{n_{tp}+n_{fn}}$$

$$\mathrm{False}\;\mathrm{Positive}\;\mathrm{Rate}=\frac{n_{fp}}{n_{tn}+n_{fp}}$$

where $n_{tp}$ indicates the total number of segments whose classes are positive and which are correctly recognized by our proposed and trained system. $n_{fn}$ indicates the total number of segments whose classes are positive, but which are wrongly recognized as negative by our proposed and trained system. $n_{fp}$ indicates the total number of segments whose classes are negative, but which are wrongly recognized as positive by our proposed and trained system. $n_{tn}$ indicates the total number of segments whose classes are negative and which are correctly recognized by our proposed and trained system.

We can make three main observations here. Firstly, as presented in Figure 20 and Figure 21, MLP outputs the best recognition performance, namely 99.0% TPR of recognizing the hand-changing process, 0.6% FPR of recognizing the activities of daily life and 12 misrecognition segments in total. RF achieves the second best recognition performance, that is 98.5% TPR of recognizing the hand-changing process, 0.9% FPR of recognizing the activities of daily life and 15 misrecognition segments in total. Secondly, NB yields the worst recognition performance, namely, 69 misrecognition segments in total, among which 58 segments of the activities of daily life are wrongly recognized as segments of the hand-changing process. k-NN provides the second worst recognition performance, that is 48 misrecognition segments in total, among which 24 segments of passing the smartphone from the right hand to the left hand are wrongly recognized as segments of passing the smartphone from the left hand to the right hand, and the other 16 segments are wrongly recognized just in the opposite case. Thirdly, as reported in Figure 22, we only pay attention to the time consumption of the test process, because the training process can be accomplished offline. k-NN is the most time-consuming for testing our experimental dataset, 1000 labeled segments of the hand-changing process and 130,050 labeled segments generated by all kinds of activities in seven days of daily life, which spends 2896.68 s. NB and MLP spend 11.18 s and 6.35 s, respectively. DT and RF are the least time-consuming, which can both complete the whole test process within 3.0 s.

In summary, RF and MLP achieve almost similar recognition performance and consume almost similar test time, but MLP is much more complicated than RF, so we finally decide to employ RF to construct the recognizer in this paper.

##### Dimension Reduction Algorithms

**Figure 23:**
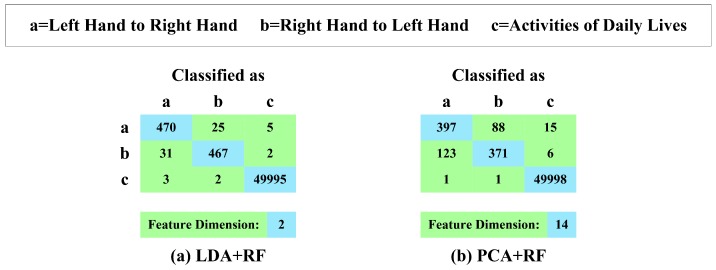
Confusion matrices and feature dimensions of RF along with LDA and PCA.

**Figure 24:**
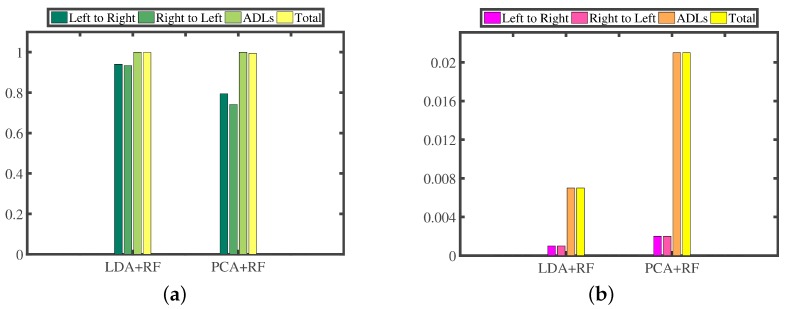
TPR (**a**) and FPR (**b**) of RF along with LDA and PCA.

Then, we construct the recognizer based on RF; in addition, we exploit PCA and LDA to reduce the dimension of features. We conduct a series of 10-fold cross-validation experiments on our experimental dataset, which contains 500 hand-changing processes performed by passing the smartphone from the left hand to the right hand, 500 hand-changing processes performed by passing the smartphone from the right hand to the left hand and 50,000 segments generated by all kinds of activities in four days of daily life. Figure 23 presents the confusion matrices; Figure 24a illustrates the TPR; and Figure 24b highlights the FPR of RF along with LDA and PCA.

We can observe that the combination of RF and LDA achieves better recognition performance, namely 93.9% precision, 93.7% TPR of recognizing the hand-changing process, 0.7% FPR of recognizing the activities of daily life and 68 misrecognition segments in total, which also reduces the features to two dimensions. The combination of RF and PCA yields worse recognition performance, that is 78.5% precision, 76.8% TPR of recognizing the hand-changing process, 2.1% FPR of recognizing the activities of daily life and 234 misrecognition segments in total, and it reduces the features to 14 dimensions.

##### Time Consumption of Every Computational Step

Next, we conduct the recognition experiment on our dataset 10 times and compute the average time consumption for every segment, as shown in Table 7, which is specific to every step. We can observe that the segment-level feature extraction is the most time consuming, and the acquisition of raw accelerometer and gyroscope data along with the segmentation of raw data into segments is the second most time consuming. The remaining steps almost do not consume time. For every segment, the whole recognition process can be accomplished within about 6.86 ms.

#### 3.6.2. Recognition Performance of DTW

Here, we give the results of our experiments, including three parts. In the first part, we present our experimental dataset. Then, we study the recognition performance in the second part. Finally, we demonstrate the time consumption of the DTW in the recognition process for every segment.

##### Experimental Dataset

As shown in Table 8, our experimental dataset contains two categories, namely template library and test set. The template library is composed of 20 hand-changing processes performed by passing the smartphone from the left hand to the right hand and 20 hand-changing processes performed by passing the smartphone from the right hand to the left hand. These segments form the *T* in Algorithm 1. The test set consists of 500 hand-changing processes performed by passing the smartphone from the left hand to the right hand, 500 hand-changing processes performed by passing the smartphone from the right hand to the left hand and 50,000 segments generated by all kinds of activities in four days of daily life.

##### Experimental Results

**Figure 25:**
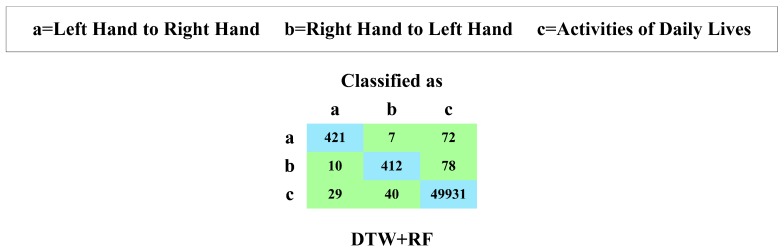
Confusion matrix of RF along with DTW.

**Figure 26:**
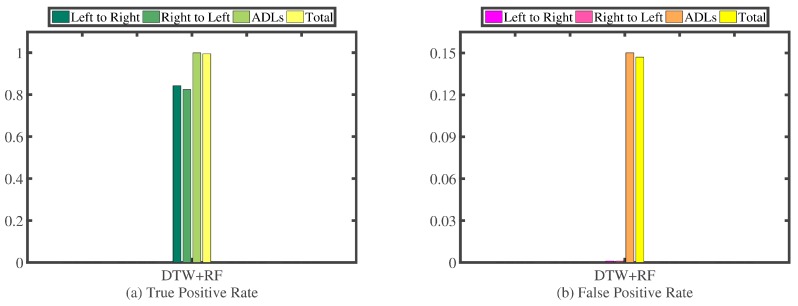
TPR (**a**) and FPR (**b**) of RF along with DTW.

In this paper, we innovatively exploit the recognition performance of RF utilizing the DTW distances between a particular segment and every segment in the template library, as features. We conduct a series of 10-fold cross-validation experiments on our experimental dataset presented in Table 8. Figure 25 presents the confusion matrix; Figure 26a illustrates the TPR; and Figure 26b highlights the FPR.

We can observe that the combination of RF and DTW achieves 83.3% TPR of recognizing the hand-changing process, 15.0% FPR of recognizing the activities of daily life and 236 misrecognition segments in total.

##### Time Consumption of DTW

We conduct the DTW to calculate the distances as features on our dataset presented in Table 8 10 times and compute the average time consumption for every segment, as shown in Table 9. We can observe that, for every segment, the DTW can be accomplished within about 233.51 ms. According to Table 7, Step 3, the segment-level feature extraction only consumes 4.53 ms. The former is 52-times as long as the latter. Due to the poor recognition performance and excessive time consumption, we eventually do not adopt the DTW in our system.

## 4. Related Work

In this section, we describe three areas of related work. In the first category, there are some smartphone touchscreen-based papers, mainly for authentication, which are relevant to our first system in this paper. In the second category, we give several smartphone accelerometer and gyroscope-based papers, mainly aiming at human activity recognition, which are relevant to our second system. In the third category, we provide some smartphone user interface adjustment-related papers.

### 4.1. Touch Behavioral Biometrics-Based Authentication

In recent years, there has been some smartphone-based biometric authentication work. Sae-Bae et al. [41] presented an authentication mechanism using multi-touch traces, and then, they defined a comprehensive five-finger touchscreen trace set that makes use of biometric information, such as hand size and finger length. Shahzad et al. [42] defined 10 kinds of specific touchscreen traces in their paper and proposed an authentication scheme for the secure unlocking of touchscreen devices using these touchscreen traces. There is some other touch feature-based identification-related work, e.g., [43,44,45]. Recently, some identification-related research work focused on using not only the touchscreen, but also smartphone-equipped sensors such as the accelerometer and gyroscope, to enrich features that can be used when identifying. GripSense [46] recognizes four kinds of hand postures, leveraging both smartphone touchscreens and inertial sensors (accelerometers, gyroscopes) and achieves 84.3% accuracy. Frank et al. [47] proposed an authentication scheme using 30 features, including touch features and inertial sensors features. However, the overfitting problem becomes more severe when too many features are used, which is called the curse of dimensionality. In their work, the EERwas approximately 13% when deciding with a single stroke. There are some other related works, e.g., [48,49,50,51,52,53].

### 4.2. Human Activity Recognition with Inertial Sensors

Massive significant inertial sensor-based human activity recognition-related research has been conducted, some of which is implemented on smartphones. Abbate et al. [54] put forward a novel smartphone-based fall detection system, which keeps monitoring the activities of the carriers and recognizing the falls. Their work we believe is of great significance to the care of elderly people. Bo et al. [55] presented a system that can estimate the locations and calculate the traveling distances of vehicles in urban environments leveraging the inertial sensors for metropolitan localization. In [56], Dai et al. proposed a smartphone-based drunk driving detection system aiming at the warning of drinking-related dangerous driving behaviors, which is also very meaningful, because their work could save lives under some particular circumstances. Kwapisz et al. [19] described and evaluated a system that takes advantage of accelerometers incorporated in smartphones to identify the physical activities performed by the users. Bhoraskar et al. [57] presented a non-intrusive system to estimate the traffic and road condition with the help of inertial sensors along with the Global Position System. Recently, researchers also explored the wearable devices for human activity recognition [58]. Parate et al. [59] designed a system that leverages a wristband to detect smoking gestures in real time, which can be employed to help people give up smoking. Ladha et al. [60] developed a climbing assistance system that aims to provide expert assessments for climbing enthusiasts. Tapia et al. [61] proposed a real-time system that not only recognizes human activities, but also measures their intensities, innovatively exploiting heart rate monitors, besides inertial sensors. Bo et al. [62] achieved a non-intrusive system that can detect the drivers’ texting operations during driving. This system is significant for driving safety. There are still many other excellent related research works, but due to the page limitation, we will not introduce them here.

### 4.3. Smartphone User Interface Adjustment

Recently, several research efforts were made about the smartphone user interface adjustment. Song et al. [63] proposed an automatic graphical user interface adjustment method based on human face tracking. Hu et al. [64] presented an intriguing system that enables a smartphone to adjust the user interface when the user is at a slanted viewing angle. They augment the smartphone camera with a fisheye lens and then employ the face recognition technology. He et al. [65] put forward a flexible dynamic resolution scaling system for a smartphone that adopts an ultrasonic-based approach to detect the distance between the user and the touchscreen, at a low power cost. Komine et al. [66] investigated how the size of the command button affects the physical performance of a user and the psychological perception of a smartphone. Alonso et al. [67] found that the graphical user interface is not specifically tailored for different smartphones with touchscreens. They conducted a user study and provided evidence that it is important to tailor the graphical user interface to make good use of the display size of a given smartphone. Several latest large screen smartphones and applications also support the function of user interface adjustment in order to improve user experience. The smartphone Smartisan [68] lets the user select the dominant hand when the smartphone boots up for the first time and allows the user to set the positions of the back button and the menu button according to his or her operation habit. The Baidu input method [69] supports single hand mode. The user can make the interactive interface shrink proportionally and gather on the side of the current operating hand by clicking a particular button. The iPhone 6 [1] user can make the whole smartphone interactive interface descend by tapping the home button twice, so the buttons on the top of the smartphone screen can be reached. However, this approach also makes the same operation that needs only one click originally now need three clicks. The interactive interface of Samsung Note 3 [3] can be shrunk in proportion and be placed on a corner of the smartphone screen, but apparently, this practice conflicts with the original intention of using a large screen smartphone. These state-of-the-art user interface adjustment functions all require the user’s intervention, such as one step or many steps of selecting operation, so these functions are impractical when the user changes the operating hand frequently. According to our research, all functions mentioned above can be achieved automatically or improved.

## 5. Discussion

### 5.1. Smartphone User Interface Adjustment

In this paper, we have implemented two systems on smartphones, which can recognize the current operating hand and the hand-changing process. After that, the user interface should be adjusted to make the smartphones easier to operate, according to the current operating hand. We have developed a demonstration application to illustrate how the whole proposed system works. On the one hand, we do not have the permission and approach to change the user interfaces of other applications, especially those developed by commercial software companies, which are protected by copyrights. On the other hand, there are hundreds of thousands of applications in the world, and for different applications, even different pages in the same application, the numbers, attributes (sensitive or should be convenient to reach) and layouts of buttons may be totally different. Accordingly, the adjustment strategies (for example, when the current operating hand has been changed from the left to the right, this button should be moved from here to there) will be totally different, for different applications. Therefore, in this paper, we did not provide the specific adjustment strategies, for every application, because that is not realistic, as mentioned before. We proposed the idea of user interface adjustment according to the current operating hand and have implemented a completely feasible technical solution. Actually, when our solution is authentically adopted in the future, we prefer it to be a port that will be integrated in the smartphone operating systems and can be provided to the software engineers directly. The remaining job, that is the user interface adjustment, is less technical, but requires more professional artistic accomplishment. 

### 5.2. Energy Consumption Analysis

#### 5.2.1. Recognition of the Current Operating Hand

There are three steps that mainly cause energy consumption [70] in the current operating hand recognition process. The first step is the acquisition of touchscreen traces. The lightening of the touchscreen will consume a certain amount of energy. However, even if our system does not exist, when people normally use smartphones, this part of the energy will still be consumed anyway. We just incidentally employ these traces generated during the users’ smartphone operation processes for recognition. Therefore, the energy consumption of the first step can be ignored. The second step is the feature extraction. As shown in Table 2, all of these features, such as length, velocity and displacement, are economical and can be easily calculated. The energy consumption of calculating these features is negligible, compared to other smartphone applications, such as the video processing in an exquisite racing or shooting game. The third step is the classification. The training process of the classifier is offline. Therefore, its energy consumption can be ignored. Then, based on the existing random forest classifier, the smartphone conducts the classification, which will not use too much energy, because the random forest classifier is extremely lightweight.

#### 5.2.2. Recognition of the Hand-Changing Process

As for the recognition of the hand-changing process, there are four steps that mainly cause energy consumption. The first step is the acquisition of the accelerometer and gyroscope data. The power of the accelerometer is about 96 mW [71]. The power of the gyroscope is about 153 mW [72]. They are both low cost. Therefore, the energy consumption of the first step is only at about the thousand-Joule-level per hour. The second step is similar to the second step of the current operating hand recognition process. Therefore, we will not analyze it again here. The third step is the dimension reduction of the features. The training process of the linear discriminant analysis is offline. Then, the smartphone conducts only two linear transformations to transform high dimensional features into low dimensional features. Therefore, the energy consumption of the third step can be ignored. The fourth step is the same as the third step of the current operating hand recognition process. Therefore, we will not analyze it again here.

#### 5.2.3. Another Related Paper

Hao et al. [73] proposed a sleep quality monitoring system, which conducts the similar processing steps as our systems. The first step is the acquisition of audio clips from the smartphone built-in microphone. The power of the microphone is about 329 mW [71], which is greater than the sum of the accelerometer and gyroscope. Then, the smartphone extracts several efficient features. In the third step, the smartphone utilizes a pre-trained decision tree classifier to detect the events that are related to sleep quality. The whole energy consumption of their system is about 4% of the battery capacity per hour.

## 6. Conclusions

User experience can be significantly improved if the smartphones are able to recognize the current operating hand, detect the hand-changing process and then adjust the user interfaces subsequently. In this paper, we proposed two novel systems. The first one leverages the user-generated touchscreen traces for the recognition of the current operating hand, and the second one utilizes the accelerometer and gyroscope data of all kinds of activities in the user’s daily life for the detection of the hand-changing process. These two systems are based on two supervised classifiers, which are constructed from several refined touchscreen trace, accelerometer and gyroscope features. We have implemented our systems on Android-based smartphones and conducted a series of experiments. Evaluation results demonstrate that our proposed systems can both recognize the current operating hand and detect the hand-changing process accurately for different users. To the best of our knowledge, this is the first paper that has proposed the idea of smartphone user interface automatic adjustment and gave a complete and feasible technical solution using touchscreen traces and accelerometer-gyroscope data.

## Figures and Tables

**Table 1:** Features extracted from touchscreen trace data.

**Length Features**	
1	Total Length
**Velocity Features**	
3	Maximum and Average Velocity, Standard Deviation of Velocity
**Displacement Features**	
4	Total and Maximum X-Displacement, Total and Maximum Y-Displacement
**Shape Features**	
4	Root Mean Squared Error, Maximum and Average Curvature, Curve Convex Orientation

**Table 2:** Computation time (Avg. ± SD) of every feature vector.

Features		Time
Length	Total	(0.046 ± 0.011) ms
Velocity	Maximum	(0.088 ± 0.040) ms
	Average	
	Standard Deviation	
Displacement	Total X	(0.037 ± 0.012) ms
	Maximum X	
	Total Y	
	Maximum Y	
Shape	RMSE	(3.834 ± 0.367) ms
	Maximum Curvature	(13.834 ± 1.005) ms
	Average Curvature	
	CCO	(0.007 ± 0.002) ms
Total		(16.867 ±1.553) ms

**Table 3:** Classification performance of different classifiers.

	Precision	Recall	F1	AUC
DT	92.2%	91.8%	91.8%	0.975
RF	95.6%	95.6%	95.6%	0.990
NB	82.4%	71.5%	72.8%	0.919
MLP	96.9%	96.8%	96.8%	0.999
k-NN	88.1%	88.2%	88.2%	0.951

**Table 4:** Features extracted on accelerometer data. Abs, absolute value.

Accelerometer Data-Based Features		
Time	4	All-Max, All-Mean, All-Min, All-Range
	3	AbsX-Max, AbsX-Mean, X-Range
	5	AbsY-Mean, AbsY-Min, Y-Max, Y-Mean, Y-Min
	5	AbsZ-Max, AbsZ-Mean, Z-Max, Z-Mean, Z-Range
Statistics	2	X-Kurtosis, Y-Kurtosis
Frequency	2	All-DC Amplitude, All-Peak Amplitude
	3	X-Centroid, X-Decrease, X-Roll-Off
	7	Y-Centroid, Y-DC Amplitude, Y-Decrease
		Y-Flux, Y-Peak Amplitude, Y-Roll-Off, Y-Spread
	5	Z-DC Amplitude, Z-Decrease, Z-Flux, Z-Peak Amplitude, Z-Spread

**Table 5:** Features extracted on gyroscope data.

Gyroscope Data-Based Features		
Time	2	All-Max, All-Range
	3	AbsY-Max, Y-Max, Y-Range
	1	Z-Mean
Statistics	2	X-Kurtosis, Y-Kurtosis
Frequency	1	All-Peak Amplitude
	4	X-Centroid, X-Decrease, X-Roll-Off, X-Spread
	3	Y-Centroid, Y-Roll-Off, Y-Spread
	2	Z-DC Amplitude, Z-Decrease

**Table 6:** Experimental dataset for recognizing the hand-changing process employing pattern recognition algorithms.

Activities	Total
Left Hand to Right Hand	500 times
Right Hand to Left Hand	500 times
Activities of Daily Life	7 days
(Walk, Run, Go Upstairs and so on)	

**Table 7:** Time consumption (Avg. ± SD) of every step in the recognition process for every segment.

Step	Computational Process		Time
1–2	Acquisition of Raw		(2.33 ± 0.09) ms
	Accelerometer and Gyroscope Data		
	Segmentation of		
	Raw Data into Segments		
3	Segment-Level Feature Extraction		(4.53 ± 0.04) ms
4	Dimension Reduction	Train	∼0 ms
	(LDA)	Test	
6	Recognition Algorithm	Train	∼0 ms
	(RF)	Test	
Total			(6.86 ± 0.11) ms

**Table 8:** Experimental dataset for recognizing the hand-changing process employing DTW.

Category	Activities	Total
Template Library	Left Hand to Right Hand	20 times
	Right Hand to Left Hand	20 times
Test Set	Left Hand to Right Hand	500 times
	Right Hand to Left Hand	500 times
	Activities of Daily Lives	4 days
	(Walk, Run, Go Upstairs and so on)	

**Table 9:** Time consumption (Avg. ± SD) of DTW in the recognition process for every segment.

Step	Computational Process	Time
5	Segment-Level Feature Extraction (DTW)	(233.51 ± 0.61) ms

## Abbreviations

The following abbreviations are used in this manuscript:

TPR	True Positive Rate
FPR	False Positive Rate
DT	Decision Tree
RF	Random Forest
NB	Naive Bayes
MLP	Multi-Layer Perceptron
k-NN	k-Nearest Neighbors
LDA	Linear Discriminant Analysis
PCA	Principal Component Analysis
DTW	Dynamic Time Warping
